# Screening and Identification of the Biomarkers Applied for the Evaluation of Acute and Chronic Thermal Tolerance Ability in Largemouth Bass (*Micropterus salmoides*)

**DOI:** 10.3390/ani14101435

**Published:** 2024-05-11

**Authors:** Ming Li, Jinxing Du, Shengjie Li, Tao Zhu, Caixia Lei, Hanwei Yan, Hongmei Song

**Affiliations:** 1 Key Laboratory of Tropical and Subtropical Fishery Resource Application and Cultivation, China Ministry of Agriculture, Pearl River Fisheries Research Institute, Chinese Academy of Fisheries Sciences, Guangzhou 510380, China; lliming6666@163.com (M.L.); ssjj@163.com (S.L.); zhutao@cau.edu.cn (T.Z.); leicaixia0703@sina.com (C.L.); yanhanwei0128@163.com (H.Y.); 2 College of Fisheries and Life Science, Shanghai Ocean University, Shanghai 201306, China

**Keywords:** thermal stress, cortisol, antioxidant enzymes, glucose metabolism-related enzymes, *hsp70/90*

## Abstract

**Simple Summary:**

Two subspecies of largemouth bass (*Micropterus salmoides*, LMB) with different thermal tolerance, northern largemouth bass (NLMB) and Florida largemouth bass (FLMB), were subjected to acute and chronic thermal stress at 33 °C. Then, variations of 12 candidate biomarkers between NLMB and FLMB were analyzed. Compared to NLMB, FLMB exhibited a lower plasma cortisol level and a higher expression of *hsp90* under acute thermal stress. Additionally, lower expression of *hsp70* in FLMB was observed under chronic thermal stress. The differences in plasma cortisol levels and *hsp* expression represent variations in thermal tolerance between the two subspecies of LMB, providing valuable information for the identification and breeding of LMB varieties with better thermal tolerance in the future.

**Abstract:**

Affected by the continuously rising temperature, thermal stress leads to a delinked growth rate and resistance to stress in cultured largemouth bass (*Micropterus salmoides*, LMB) in China. Identification of LMB with better thermal resistance will benefit the breeding of new varieties. However, there has been limited reporting on the evaluation to identify LMB with better thermal resistance. LMB consists of the northern LMB (*Micropterus salmoides salmoides*, NLMB) and the Florida LMB (*Micropterus salmoides floridanus*, FLMB). Due to their different geographical distributions, it has been suggested that FLMB exhibit better thermal resistance compared to NLMB. In this study, NLMB and FLMB were subjected to thermal stress for 3 h (acute) and 60 d (chronic) at 33 °C, respectively. Subsequently, the variations of 12 candidate biomarkers between NLMB and FLMB were analyzed. Exposure to acute thermal stress significantly increased plasma cortisol, blood glucose, and lactate levels; activities of superoxide dismutase (SOD), glutathione peroxidase (GPX), catalase (CAT), glucose kinase (GK), pyruvate kinase (PK), lactate dehydrogenase (LDH), and glucose 6 phosphatase (G6Pase); and the expressions of *hsp70* and *hsp90* in both NLMB and FLMB (*p* < 0.05). Compared to NLMB, FLMB exhibited a lower plasma cortisol level and a higher expression of *hsp90* under acute thermal stress (*p* < 0.05). Exposure to chronic thermal stress significantly increased plasma cortisol and blood glucose levels, as well as activities of GK, PK, LDH, and G6Pase, as well as expressions of *hsp70* and *hsp90* in both NLMB and FLMB (*p* < 0.05). Additionally, FLMB showed a lower expression of *hsp70* compared to NLMB (*p* < 0.05). In conclusion, our results showed that LMB with lower plasma cortisol level and higher expression of *hsp90* under acute thermal stress, as well as lower expression of *hsp70* under chronic thermal stress were suggested to have better thermal resistance. Our study provides valuable information for identifying and breeding LMB varieties with better thermal resistance in the future.

## 1. Introduction

The largemouth bass (*Micropterus salmoides*, LMB) is native to North America and was introduced from Taiwan to mainland China in 1983. In recent decades, it has become an important cultured fish in China, with production of 802,486 tons in 2022. The survival and growth of LMB are impacted by various biological and abiotic stresses, such as water temperature, quality, and density [1,2,3]. One of the key factors influencing the growth, feeding, energy consumption, respiration, and mobility of LMB is water temperature [2,4]. According to the 2022 China Fishery Statistical Yearbook, Guangdong, Zhejiang, and Jiangsu Provinces accounted for 80% of LMB production. However, high temperatures have been occurring frequently in these regions in recent years. For instance, it has been reported that the maximum surface water temperature in LMB culture ponds in Sichuan Province reached 34.4 °C during the summer seasons from 2017 to 2019 [5]. Under thermal stress, LMB experienced problems such as decreased food intake, declined growth rate, and reduced stress resistance. Identifying LMB with better thermal resistance will benefit the breeding of LMB varieties with improved stress tolerance.

LMB have originally been considered to comprise two subspecies, the northern LMB (*Micropterus salmoides salmoides*, NLMB), distributed in most central and eastern parts of America, northeast Mexico, and southeast areas of Canada; and the Florida LMB (*Micropterus salmoides floridanus*, FLMB), found on the Florida peninsula [6,7]. Due to their geographical distribution differences, it has been reported that FLMB are more resistant to thermal stress than NLMB. For instance, the chronic caloric maximum of FLMB was 39.2 ± 0.64 °C, which was 37.3 ± 0.60 °C in NLMB [8]. Median lethal 24 h temperatures for 0–3 d hatched larvae averaged 32.1 °C for NLMB and 32.6 °C for FLMB [9]. Through morphological traits, microsatellite molecular markers [10], DNA fingerprinting [11], SNP, and InDel markers [12], we confirmed that the cultured LMB in China belong to the NLMB classification. However, the mechanism underlying thermal resistance differences between the two subspecies remains unknown.

Stress responses of fish can be roughly divided into primary, secondary, and tertiary stages [13,14]. Primary responses include initial neuroendocrine reactions that activate brain regions and cause a large-scale release of corticosteroids and catecholamines. However, unlike the rapid release of catecholamines from chromaffin cells, cortisol synthesis and release from interrenal cells have a lag time of several minutes. Therefore, circulating levels of cortisol are commonly used as a stress indicator of fish [15]. Secondary responses are typically described as the manyfold immediate actions and effects of these hormones at the blood and tissue level, resulting in various hematological and biochemical parameters, including blood glucose, lactate, antioxidant enzymes, glucose metabolism-related enzymes, and heat-shock stress protein expression. Tertiary responses extend to the welfare of fish, encompassing their survival, growth, reproductive capabilities, and behavior [14,16]. Therefore, assessment of the hematological and biochemical parameters will be a useful tool for investigating the ability of fish to adapt to the environment [17,18]. In LMB, it has been reported that blood glucose levels significantly increased after acute thermal stress at 33 °C for 6 h [19]. Similar variations have been observed in activities of superoxide dismutase (SOD), glutathione peroxidase (GPX), and catalase (CAT) [20], as well as expression of *hsc70* and *hsp70* in the liver and gill tissues [21]. However, whether these candidate biomarkers are suitable for the evaluation of thermal resistance in LMB remains to be further explored.

As a warm-water fish, the LMB is suggested to have an optimal growth temperature of 20–25 °C [22]. Water temperatures exceeding 32 °C inhibit LMB growth, while temperatures exceeding 34 °C cause death [9,20]. Therefore, the experimental treatment temperature in this study was set at 33 °C. After being treated with acute (3 h) and chronic (60 d) thermal stress, we analyzed variations in blood parameters (cortisol, blood glucose, and lactate), antioxidant enzymes activities (SOD, GPX, and CAT), glucose metabolism-related enzymes activities (glucose kinase (GK), pyruvate kinase (PK), lactate dehydrogenase (LDH), and glucose 6 phosphatase (G6Pase)), as well as expressions of *hsp70* and *hsp90* between NLMB and FLMB. Through these experiments, the biomarkers which revealed significant differences between NLMB and FLMB under thermal stress will be used to identify the LMB with better thermal tolerance abilities. Our study provides valuable information for the breeding of new varieties of LMB in the future.

## 2. Materials and Methods

### 2.1. Experimental Materials and Experimental Design

NLMB and FLMB were obtained from Guangdong Liangshi Aquatic Seed Industry Co., Ltd. (Foshan, China), with an average body weight of 20.64 ± 2.13 g and 20.20 ± 1.35 g, respectively. A total of 180 NLMB and 180 FLMB were randomly divided into 6 replicates, with 30 fish in each replicate. Before the thermal stress treatments, these fish were held in 12 circular 125 L glass tanks with a filtration system (equipped with a heating system and a water flow rate of 5 L/min; 14 h light: 10 h darkness) for temporary rearing at 25 °C for 1 week. Two groups were involved in the acute thermal stress treatments: the short-term 25 °C group (S25) and the short-term 33 °C group (S33). To achieve the experimental treatment temperature, the water temperatures in the acute thermal stress treatments were adjusted at a rate of 0.1 °C/min. The chronic thermal stress treatments included two groups: the long-term 25 °C group (L25) and the long-term 33 °C group (L33). The water temperature in the chronic thermal stress treatments was adjusted at the rate of 1 °C/d until the experimental treatment temperature was reached. Additionally, the NLMB and FLMB were maintained at 33 °C for 60 days. During this period, the LMB were fed a commercial artificial diet (50% crude protein, Tianma Technology Group Co., Ltd., Fuqing, China) to apparent satiation twice daily (8:00–9:00 and 17:00–18:00) The water was cleaned once per week.

### 2.2. Sample Collection

After being kept at the experimental treatment temperatures for 3 h, the samples were collected from the groups that received acute thermal stress treatment. After being maintained at the experimental treatment temperatures for 60 d, the samples were collected from the groups that received chronic thermal stress treatment. Five fish were randomly selected in each replicate and anesthetized with MS-222 (Tianjin, China). Blood samples were immediately collected from the tail vein using a syringe, stored in 1.5 mL centrifuge tubes at 4 °C for 2 h, and then centrifuged at 4000 rpm/min for 10 min. The supernatant was collected and transferred to a 1.5 mL centrifuge tube, which was stored at −80 °C. Liver samples were excised and stored at −80 °C.

### 2.3. Determination of Cortisol, Blood Glucose, Lactate, and Activities of Enzymes

Serum cortisol and lactate were determined using commercial kits (Nanjing Jiancheng Bioengineering Institute, Nanjing, China) according to the manufacturer’s instructions. Blood glucose was measured using a blood glucose meter (ACCU-CHEK Performa, Roche, Shanghai, China). Activities of antioxidant enzymes (SOD, GPX, CAT), and glucose metabolism-related enzymes (GK, PK, LDH, G6Pase) were determined using commercial kits (Shanghai Preferred Bioscience Being Co., Ltd., Shanghai, China) according to the manufacturer’s instructions.

### 2.4. Expression Analysis of Hepatic hsp Genes

Sequences of *hsp70* (*MN121693.1*) and *hsp90* (*XM_038705070.1*) were obtained according to the reference genome of LMB (GenBank: GCA_019677235.1). Total RNA was extracted using TRIzol Reagent (Invitrogen, Shanghai, China) according to the manufacturer’s instructions. RNA integrity and quantity were determined using an Agilent 2100 Bioanalyzer (Agilent, Shanghai, China). The synthesis of cDNA was performed with a ToloScript All-in-one RT Easy Mix for qPCR kit (Shanghai, China) following the manufacturer’s instructions. qRT-PCR was conducted using SYBR Green Premix ExTaq (Takara, Dalian, China) in a CFX96 real-time PCR system (Bio-Rad, Hercules, CA, USA). qRT-PCR was performed in a 20 μL reaction mixture including 10 μL SYBR Premix ExTaq™ II (2×), 0.5 μL of each primer (10 μM), 1 μL of cDNA, and 8.0 μL of ddH2O. The PCR procedure was as follows: 95 °C for 2 min, followed by 40 cycles of 95 °C for 10 s and 60 °C for 10 s, a 0.5 °C/5-s incremental increase from 58 to 95 °C, and 30s elapse time for each cycle. The expression level was estimated using the 2^−ΔΔCt^ method [23]. *β-actin* was used as the internal reference gene to normalize the gene expression level (Table 1). Five biological and three technical replicates were used for each gene.

### 2.5. Data Analysis

Experimental data were expressed as mean ± standard deviation (Mean ± SD). Since there was only one factor (i.e., temperature or subspecies) in our experimental designs, an independent sample *t*-test was performed to examine the effects of physiological and molecular parameters. The experimental data were processed using the software SPSS.26, and a significance level of *p* < 0.05 was used as the criterion for statistical significance.

## 3. Results

### 3.1. Effects of Acute and Chronic Thermal Stress on the Plasma Cortisol, Blood Glucose, and Lactate Levels

**Figure 1:**
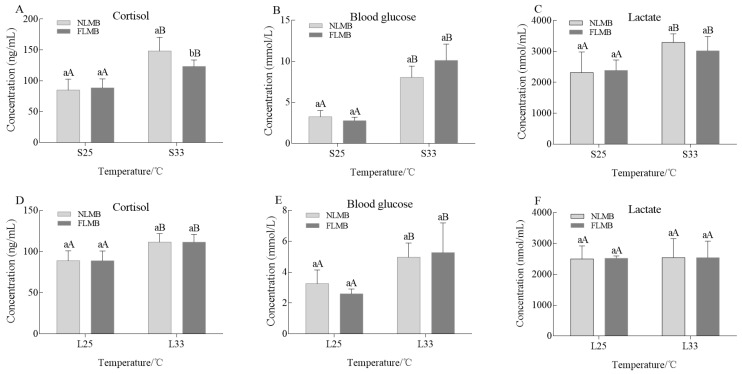
Effects of acute and chronic thermal stress on the variations of plasma cortisol, blood glucose, and lactate levels in NLMB and FLMB (*n* = 15). Note: (**A**–**C**) were the variations in plasma cortisol, blood glucose, and lactate levels under acute thermal stress, respectively. (**D**–**F**) were variations in plasma cortisol, blood glucose, and lactate levels under chronic thermal stress, respectively. Different lowercase letters above the bars show significant differences in the same groups, and different uppercase letters above the bars show significant differences in the same subspecies (*p* < 0.05).

The variations in plasma cortisol, glucose, and lactate levels of NLMB and FLMB under acute thermal stress are shown in Figure 1A–C. There were no significant differences in plasma cortisol, blood glucose, and lactate levels between NLMB and FLMB in the S25 group. Compared to the S25 group, plasma cortisol, blood glucose, and lactate levels in the S33 group were significantly increased in both NLMB and FLMB (*p* < 0.05). The plasma cortisol level of NLMB was significantly higher than that of FLMB in the S33 group (*p* < 0.05). However, there were no significant differences in blood glucose and lactate levels between NLMB and FLMB in the S33 group.

The variations in plasma cortisol, blood glucose, and lactate levels of NLMB and FLMB under chronic thermal stress are shown in Figure 1D–F. There were no significant differences in levels of plasma cortisol, blood glucose, or lactate between NLMB and FLMB in the L25 group. Compared to the L25 group, plasma cortisol and blood glucose levels in the L33 group were significantly increased in both NLMB and FLMB (*p* < 0.05). However, there was no significant difference in lactate levels between the L25 and L33 groups. Additionally, there were no significant differences in plasma cortisol, blood glucose, or lactate levels between NLMB and FLMB in the L33 group.

### 3.2. Effects of Acute and Chronic Thermal Stress on the Activities of Antioxidant Enzymes in Liver Tissues

**Figure 2:**
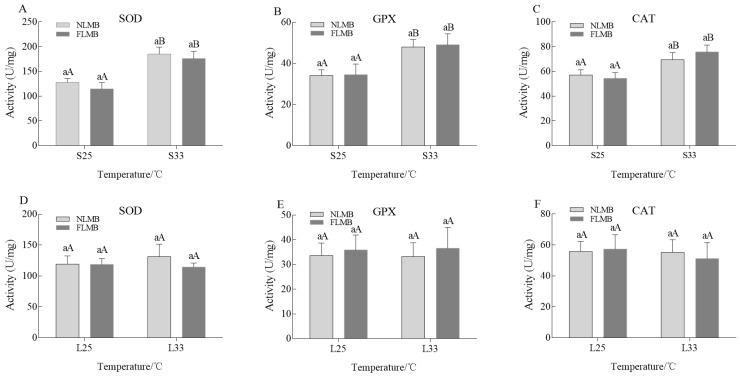
Effects of acute and chronic thermal stress on the activity variations of antioxidant enzymes in liver tissues of NLMB and FLMB (*n* = 15). Note: (**A**–**C**) were the activity variations of SOD, GPX, and CAT enzymes under acute thermal stress, respectively. (**D**–**F**) were the activity variations of SOD, GPX, and CAT enzymes under chronic thermal stress, respectively. Different lowercase letters above the bars show significant differences in the same groups, and different uppercase letters above the bars show significant differences in the same subspecies (*p* < 0.05).

The activity variations of SOD, GPX, and CAT enzymes in the livers of NLMB and FLMB under acute thermal stress are shown in Figure 2A–C. There were no significant differences in the activities of SOD, CAT, or GPX between NLMB and FLMB in the S25 group. Compared to the S25 group, the activities of SOD, CAT, and GPX enzymes in the S33 group were significantly increased in both NLMB and FLMB (*p* < 0.05). However, there were no significant differences in the activities of SOD, CAT, and GPX between NLMB and FLMB in the S33 group.

The activity variations of SOD, GPX, and CAT enzymes in the livers of NLMB and FLMB under chronic thermal stress are shown in Figure 2D–F. There were no significant differences in the activities of SOD, GPX, or CAT enzymes between NLMB and FLMB in the L25 group. Compared to the L25 group, there were no significant differences in the activities of SOD, GPX, or CAT enzymes in the L33 group. Additionally, there were no significant differences in the activities of SOD, CAT, or GPX between NLMB and FLMB in the L33 group.

### 3.3. Effects of Acute and Chronic Thermal Stress on the Activities of Glucose Metabolism-Related Enzymes in Liver Tissues

**Figure 3:**
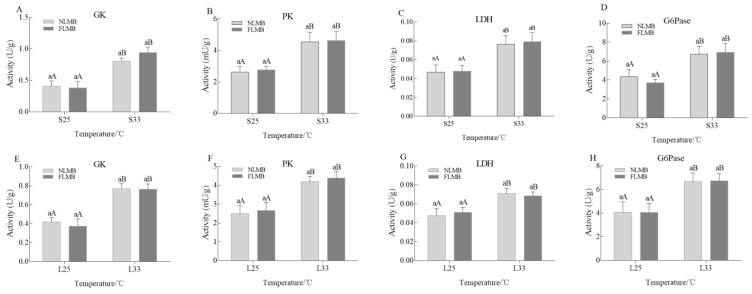
Effects of acute and chronic thermal stress on the activity variations of glucose metabolism-related enzymes in liver tissues of NLMB and FLMB (*n* = 15). Note: (**A**–**D**) were the activity variations of GK, PK, LDH, and G6Pase enzymes under acute thermal stress, respectively. (**E**–**H**) were the activity variations of GK, PK, LDH, and G6Pase enzymes under chronic thermal stress, respectively. Different lowercase letters above the bars show significant differences in the same groups, and different uppercase letters above the bars show significant differences in the same subspecies (*p* < 0.05).

The activity variations of GK, PK, LDH, and G6Pase enzymes in the liver of NLMB and FLMB under acute thermal stress are shown in Figure 3A–D. There were no significant differences in the activities of GK, PK, LDH, or G6Pase enzymes between NLMB and FLMB in the S25 group. Compared to the S25 group, the activities of GK, PK, LDH, and G6Pase enzymes in the S33 group were significantly increased in both NLMB and FLMB (*p* < 0.05). However, there were no significant differences in the activities of GK, PK, LDH, or G6Pase enzymes between NLMB and FLMB in the S33 group.

The activity variations of GK, PK, LDH, and G6Pase enzymes in the liver of NLMB and FLMB under chronic thermal stress are shown in Figure 3E–H. There were no significant differences in the activities of GK, PK, LDH, or G6Pase enzymes between NLMB and FLMB in the L25 group. Compared to the L25 group, activities of GK, PK, LDH, and G6Pase enzymes in the L33 group were significantly increased in both NLMB and FLMB (*p* < 0.05). However, there were no significant differences in activities of GK, PK, LDH, or G6Pase enzymes between NLMB and FLMB in the L33 group.

### 3.4. Effects of Acute and Chronic Thermal Stress on the Expressions of hsp70 and hsp90 in Liver Tissues

**Figure 4:**
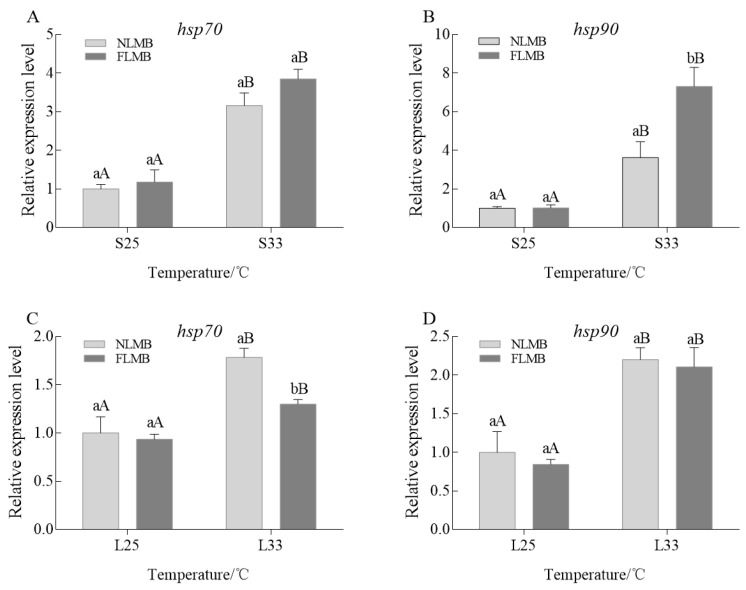
Effects of acute and chronic thermal stress on the expression variations of *hsp70* and *hsp90* in liver tissues of NLMB and FLMB (*n* = 15). Note: (**A**,**B**) were the expression variations of *hsp70* and *hsp90* under acute thermal stress, respectively. (**C**,**D**) were the expression variations of *hsp70* and *hsp90* under chronic thermal stress, respectively. Different lowercase letters above the bars show significant differences in the same groups, and different uppercase letters above the bars show significant differences in the same subspecies (*p* < 0.05).

The expression variations of *hsp70* and *hsp90* in the livers of NLMB and FLMB under acute thermal stress are shown in Figure 4A,B. There were no significant differences in expression of *hsp70* or *hsp90* between NLMB and FLMB in the S25 group. Compared to the S25 group, the expression of *hsp70* and *hsp90* in the S33 group were significantly upregulated in both NLMB and FLMB (*p* < 0.05). There was no significant difference in expression of *hsp70* between NLMB and FLMB in the S33 group. However, FLMB showed a significantly higher expression of *hsp90* than NLMB did in the S33 group (*p* < 0.05).

The expression variations of *hsp70* and *hsp90* in the livers of NLMB and FLMB under chronic thermal stress are shown in Figure 4C,D. There were no significant differences in expression of *hsp70* or *hsp90* between NLMB and FLMB in the L25 group. Compared to the L25 group, the expressions of *hsp70* and *hsp90* in the L33 group were significantly upregulated in both NLMB and FLMB (*p* < 0.05). NLMB had a significantly upregulated expression of *hsp70* than FLMB in the L33 group (*p* < 0.05). However, there was no significant difference in the expression of *hsp90* between NLMB and FLMB in the L33 group.

## 4. Discussion

Under stressors, cortisol is synthesized in the interrenal cells and released into the blood [24], which contributes to protecting organisms and avoiding oxidative stress. Meanwhile, cortisol elevates metabolic levels and mobilizes reserve energy by increasing gluconeogenesis in the liver [25], which raises blood glucose level to provide sufficient energy for coping with temperature variation [26]. Additionally, acute stress enhances anaerobic respiration processes that metabolize glucose into lactate, resulting in increased energy production. Previous studies have suggested that thermal stress treatments increased the cortisol, blood glucose, and lactate levels in large yellow crocea (*Larimichthys crocea*) [27], Korean rockfish (*Sebastes schlegelii*) [28], gemstone bass (*Scortum barcoo*) [29], Chinese sturgeon (*Acipenser sinensis*) [30], and *Eleginops maclovinus* [31]. In this study, both cortisol and blood glucose levels were significantly higher in the S33 group and L33 group compared to the control group. These results indicate that corticosteroids and gluconeogenesis processes are key for LMB to cope with thermal stress. Lactate levels were significantly higher in the S33 group compared to the S25 group, while no significant difference was observed between the L25 group and the L33 group. These results may be related to differences in the duration of thermal stress treatment. Under chronic thermal stress, LMB gradually adapted to the variation temperatures, leading to a dynamic balance between lactate synthesis and decomposition. Moreover, FLMB had lower cortisol levels compared to NLMB in the S33 group, which was consistent with its better thermal resistance [8,9]. This result suggests that LMB with lower plasma cortisol level under acute thermal stress have better thermal resistance.

Production of excessive reactive oxygen species (ROS) causes oxidative stress in fish after exposure to stressors. To maintain homeostasis, organisms increase the activities of antioxidant enzymes, such as SOD, GPX, CAT, etc., to scavenge the excessive ROS [32,33]. For instance, acute thermal stress increased the activities of SOD, GPX, and CAT enzymes in rainbow trout (*Oncorhynchus mykiss*) [34] and *Scapharca subcrenata* [35]. In this study, the activities of SOD, GPX, and CAT enzymes were increased after exposure to acute thermal stress for 3h, which was similar to the findings by Lu et al. in LMB [20]. However, the activities of SOD, GPX, and CAT enzymes had no significant differences after chronic thermal stress. These variations may be related to the duration of treatment. Under acute thermal stress, the activities of SOD, GPX, and CAT enzymes evidenced a trend of first increasing and then decreasing in pikeperch (*Sander lucioperca*) [36] and *Onychostoma macrolepi* [37], suggesting an adaptation process of fish under thermal stress. In comparison to acute thermal treatments, fish have more time to adapt to chronic thermal treatments. In pikeperch [38] and Stichopus japonicus (*Apostichopus japonicus*) [39], it was observed that the activities of antioxidant enzymes decreased to normal levels after 48 h and 12 h of chronic thermal stress, respectively. In addition, there were no significant differences regarding the activities of SOD, GPX, or CAT enzymes between NLMB and FLMB under acute or chronic thermal stress. These results suggest that variations in SOD, GPX, and CAT activities cannot be applied to evaluate the thermal resistance of LMB.

Both acute and chronic thermal stress caused an increase in the blood glucose levels of LMB. To better understand the mechanism of glucose metabolism in thermal stress, one rate-limiting enzyme (G6Pase) in the gluconeogenesis process and three main rate-limiting enzymes (GK, PK, and LDH) in the glycolysis process were selected. G6Pase hydrolyzes glucose 6-phosphate into free glucose and releases it into the bloodstream. Glucose is decomposed into pyruvate under the action of GK and PK. Pyruvate is fully oxidized under aerobic conditions, releasing carbon dioxide, but is fermented by LDH under anaerobic conditions to lactate or ethanol [40]. In this study, both acute and chronic thermal stress significantly increased the activities of G6Pase, GK, PK, and LDH enzymes. Similar findings have also been reported in other fish species. For instance, exposure to acute thermal stress significantly increased the activities of GK and PK in Gilthead Sea bream (*Sparus aurata*) [41] and European sea bass (*Dicentrarchus labrax*) [42], while chronic thermal stress increased the activity of LDH in pearl oysters (*Pinctada fucata*) [43]. The increased activity of G6Pase was consistent with the increased blood glucose level. The increased activities of GK, PK, and LDH indicated that glycolysis was the primary process for energy supplementation under thermal stress. However, there were no significant differences in the four-glucose metabolism-related enzymes between the NLMB and FLMB under acute or chronic thermal stress. These results suggest that the variations of glucose metabolism-related activities cannot be used to evaluate the thermal resistance of LMB.

*Hsp* are highly conserved proteins and play important roles in thermal tolerance and acclimation and assist cells in recovering from stress [44,45]. The various types of *hsp* are differentiated into five families based on their molecular weights. Among these families, *hsp70* translocases proteins across cellular membranes and protects neurons from apoptosis [46,47], while *hsp90* assists cortisol by transmitting signals from glucocorticoid receptors [48]. In this study, the expressions of *hsp70* and *hsp90* were significantly upregulated in the acute and chronic thermal-treated groups compared to the control groups. Similarly, many investigators have also reported that thermal stress induces upregulation of the expression of *hsp70* and *hsp90* in the liver of Siberian sturgeon (*Acipenser baeri*) [49], Mandarin fish [50], *Catla catla* [51], and white sturgeon larvae (*Acipenser transmontanus*) [52]. The upregulated expression of *hsp70* is likely a result of the apoptotic defense mechanism triggered by increased water temperature [53]. The upregulated expression of *hsp90* is likely due to an increase in cortisol secretion, which helps maintain cellular homeostasis and leads to increased expression of *hsp90* [54]. In the S33 group, the expression of *hsp90* in FLMB was significantly higher than that in NLMB. This difference in expression may have resulted from higher cortisol levels in NLMB, which inhibited *hsp90* expression [55]. The expression of *hsp70* was significantly lower in FLMB than in NLMB under chronic thermal stress, probably because FLMB is more thermally resistant, and the microvariation of *hsp70* would be able to resist thermal stress. In summary, the detection results of *hsp* expressions suggest that LMB with higher expression of *hsp90* under acute thermal stress and a lower expression of *hsp70* under chronic thermal stress have better thermal resistance.

## 5. Conclusions

In this study, two subspecies of LMB with different thermal resistance were used to screen for candidate biomarkers related to thermal stress response. Based on their variations under thermal stress, our results indicated that the FLMB, which exhibits better thermal resistance, had a lower plasma cortisol level and a higher expression of *hsp90* under acute thermal stress, as well as a lower expression of *hsp70* under chronic thermal stress compared to NLMB. These findings suggest that LMB with lower plasma cortisol level and higher expression of *hsp90* under acute thermal stress, as well as lower expression of *hsp70* under chronic thermal stress, are likely to have better thermal resistance. Our study provides valuable information for identifying and breeding LMB varieties with better thermal resistance in the future.

## Figures and Tables

**Table 1:** Primer sequences of *hsp70* and *hsp90*.

Gene Game	Primer Sequence (5′→3′)	
	Forward Primer	Reverse Primer
*hsp70 (MN121693.1)*	GCAGACGCAGACCTTCACCA	GGGAACACCACGAGGAGCAG
*hsp90 (XM_038705070.1)*	TGCGCTTCCAGACCTCCAAC	TCAGCCTCCTTCCTGCTGGT
*β-actin*	AAAGGGAAATCGTGCGTGAC	AAGGAAGGCTGGAAGAGGG

## Data Availability

The original contributions presented in the study are included in the article, further inquiries can be directed to the corresponding author.
